# Burleson County Medical Society

**Published:** 1886-04

**Authors:** 


### BURLESON COUNTY MEDICAL SOCIETY.

We learn by letter from Dr. M. M. Myers, of Krohne, that the medical profession of Burleson county met at Caldwell on 30th ult. and effected an organization auxilliary to the Texas State Medical Association, and that delegates will be sent to Dallas and St. Louis—to swell the already large representation of the Texas profession. The officers are, President, Dr. H. H. Darr. of Caldwell; Vice President, Dr. Sims; Secretary and Treasurer, Dr. M. M. Myers. Dr. Myers will represent the society at Dallas, Dr. Darr alternate. DANIEL'S TEXAS MEDICAL JOURNAL was chosen as the official organ of the society.

Thus the good work goes bravely on. Land in sight!

